# Nutritional background changes the hypolipidemic effects of fenofibrate in Nile tilapia (*Oreochromis niloticus*)

**DOI:** 10.1038/srep41706

**Published:** 2017-01-31

**Authors:** Li-Jun Ning, An-Yuan He, Dong-Liang Lu, Jia-Min Li, Fang Qiao, Dong-Liang Li, Mei-Ling Zhang, Li-Qiao Chen, Zhen-Yu Du

**Affiliations:** 1 Laboratory of Aquaculture Nutrition and Environmental Health (LANEH), School of Life Sciences, East China Normal University, Shanghai, China

## Abstract

Peroxisome proliferation activated receptor α (PPARα) is an important transcriptional regulator of lipid metabolism and is activated by high-fat diet (HFD) and fibrates in mammals. However, whether nutritional background affects PPARα activation and the hypolipidemic effects of PPARα ligands have not been investigated in fish. In the present two-phase study of Nile tilapia (*Oreochromis niloticus*), fish were first fed a HFD (13% fat) or low-fat diet (LFD; 1% fat) diet for 10 weeks, and then fish from the first phase were fed the HFD or LFD supplemented with 200 mg/kg body weight fenofibrate for 4 weeks. The results indicated that the HFD did not activate PPARα or other lipid catabolism-related genes. Hepatic fatty acid β-oxidation increased significantly in the HFD and LFD groups after the fenofibrate treatment, when exogenous substrates were sufficiently provided. Only in the HFD group, fenofibrate significantly increased hepatic PPARα mRNA and protein expression, and decreased liver and plasma triglyceride concentrations. This is the first study to show that body fat deposition and dietary lipid content affects PPARα activation and the hypolipidemic effects of fenofibrate in fish, and this could be due to differences in substrate availability for lipid catabolism in fish fed with different diets.

Because of the increasing cost and the limited supply of fish meal worldwide, the use of high-fat (HF) diets is a current trend in aquaculture to play a “protein sparing effect” and reduce nitrogen excretion^1^. However, HF diets often lead to severe lipid accumulation in the tissues of farmed fish, including the liver and abdominal adipose tissue, and cause metabolic disturbances^2^,^3^,^4^. Therefore, potential regulatory mechanisms to decrease fat deposition in fish are receiving considerable attention.

Peroxisome proliferator-activated receptor α (PPARα) is a nuclear transcriptional factor and has been recognized as a master regulator of lipid metabolism, particularly lipid catabolism, in mammals^5^,^6^. A large number of naturally occurring compounds, such as free fatty acids (FFA) and their metabolites, and synthetic compounds, such as plasticizers and fibrates, are PPARα ligands and many in the latter group have been developed as commercial drugs to activate lipid catabolism^7^,^8^. For example, fibrates are very common PPARα ligands that have been used to treat coronary heart disease and hyperlipidemia for decades^9^,^10^. Of note, activation of PPARα in mammals is correlated with nutritional background. Some studies have indicated that HF diet-fed animals develop a self-protective PPARα activation mechanism to counteract excessive lipid loading^11^. Dietary supplementation with fenofibrate, a widely used PPARα ligand, may strengthen the lipid-lowering effect in HF diet-fed animals by increasing the activities and expression of a number of enzymes or genes involved in lipid catabolism^12^,^13^.

Because of the key regulatory roles of PPARα in the lipid-lowering process in mammals, PPARα and its ligands have gained increasing interest in fish. Till now, the PPARα molecules of sea bass, blunt snout bream, Japanese sea bass, red sea bream, yellow catfish, plaice, and gilthead sea bream have been cloned^14^,^15^,^16^,^17^,^18^,^19^. Moreover, some studies have reported that mammalian PPARα ligands, such as fibrates, could play lipid-lowering effects in grass carp, yellow catfish, and rainbow trout^20^,^21^,^22^. However, whether the same self-protective activating mechanism of PPARα and enhanced hypolipidemic effect of PPARα ligands exist in HF diet-fed fish remains unknown. In fact, not only effective lipid-lowering factors but also nutritional backgrounds, which would affect the application of lipid-lowering factors, are important in fish lipid studies from laboratory to practical aquaculture.

Nile tilapia (*Oreochromis niloticus*) is an important aquaculture species cultured worldwide. A number of studies have indicated that the optimal dietary lipid level in Nile tilapia is 5% to 7.4%^23^,^24^. The present study included a two-phase feeding trial (Fig. 1) to understand and characterize the possible effects of a HF diet on activation of PPARα and the hypolipidemic effects of PPARα ligands in Nile tilapia. This could also help to illustrate potential regulatory mechanisms to alleviate severe fat deposition in fish. First, juvenile Nile tilapia were fed HF (13%) or low-fat (LF) (1%) diets for 10 weeks to determine if PPARα and related lipid catabolic indices were activated by the HF diet. Then, 24 fish were selected from each group in the first phase and were fed the HF or LF diet supplemented with 200 mg fenofibrate/body weight/d for another 4 weeks to investigate the effect of nutritional background on the hypolipidemic effect of fenofibrate. This is the first study to report the effects of nutritional background on PPARα activation and its hypolipidemic function in fish.

## Results

### HF diet induces lipid deposition and changes lipid metabolism-related gene expression but does not stimulate PPARα expression

After the first phase of the feeding trial, growth, body and plasma composition, and the mRNA expression of the genes related to lipid metabolism were evaluated to obtain an overview of the systemic effects of the HF diet on lipid metabolism in tilapia (Figs 2 and 3). The 10-week HF feeding trial did not change body or liver weight (Fig. 2A,B), triglycerides (TG) concentrations in liver, muscle and adipose tissue (Fig. 2D); or plasma activities of aspartate aminotransferase (AST), alanine aminotransferase (ALT) (Fig. 2E), TG, total cholesterol (TC), FFA, low-density lipoprotein (LDL), and high-density lipoprotein (HDL) (Fig. 2F), but it did significantly increase the quantity of mesenteric fat (Fig. 2C) and plasma malondialdehyde (MDA) concentration (Fig. 2F). As shown in Fig. 3, feeding the HF diet did not change expression levels of PPARα, PPARβ, PPARγ, sterol regulatory element binding protein c (SREBP1c), carnitine palmitoyltransferase 1a/b (CPT1a/b), acyl-CoA oxidase (ACO), acetyl-CoA carboxylase beta (ACCβ) or cluster determinant 36 (CD36) in the liver; PPARα, PPARβ, CPT1a, CPT1b, ACO, or CD36 in red muscle; PPARα, CPT1a, CPT1b, ACO, ACCβ, or CD36 in white muscle; or PPARα, PPARβ and PPARγ in adipose tissue. However, it significantly decreased fatty acid-binding protein 4 (FABP4) in the liver, ACCβ and FABP4 in red muscle, FABP4 in white muscle, but increased PPARβ in white muscle; and SREBP1c, adipose triglyceride lipase (ATGL), and hormone-sensitive lipase (HSL) in adipose tissue. These results indicate that the 10-week HF feeding trial increased body lipid deposition, decreased the expression of some adipogenic genes in liver and muscle, but did not stimulate the self-protective activating mechanism of PPARα or related lipid catabolism. We also measured the mRNA expressions of some PPARα-sensitive downstream genes, including Plin2, PDK4 and Ehhadk, in different tissues, but only the Ehhadk mRNA expression was higher in HF groups than in LF group in liver (Supplemental Fig. S2A).

### Nutritional background changes the hypolipidemic effect of fenofibrate on body and plasma lipid indices

Fish fed the HF or LF diets for 10 weeks were treated with fenofibrate for 4 weeks to investigate whether nutritional background changes the hypolipidemic effect of fenofibrate. As shown in Figs 4 and 5, the 4-week fenofibrate treatment did not change body weight, the quantity of mesenteric fat (Fig. 4A,C), hepatic concentrations of glycerol, muscle and adipose tissue TG concentrations (Fig. 4E–G), or plasma FFA, LDL and MDA concentrations, as well as AST, and ALT activities in either the HF or LF groups (Fig. 5C,D,F–H), but significantly decreased the hepatosomatic index (Fig. 4B). Notably, fenofibrate significantly decreased hepatic TG and plasma TG and TC concentrations only in the HF group (Figs 4D and 5A,B). In addition, fenofibrate significantly increased plasma HDL concentration in the HF group (Fig. 5E). There were no interaction effects between the lipid level and fenofibrate treatment found in most of the body and plasma indices, except plasma TG content (Supplemental Table S3), showing lipid level and fenofibrate both regulated the plasma TG content. These results indicate that the PPARα ligand fenofibrate could play a lipid-lowering effect in liver and plasma of Nile tilapia, and this effect was affected by nutritional background.

### Fenofibrate has a hypolipidemic effect by increasing fatty acid β-oxidation

[1-^14^C] Palmitate β-oxidation was measured in liver, muscle, and adipose tissue homogenates to verify the biochemical routes of the fenofibrate hypolipidemic effects. Figure 6A–C show that fenofibrate significantly increased total fatty acid β-oxidizing activities in both groups, primarily in the liver rather than in muscle or adipose tissue. Furthermore, hepatic activity of monoamine oxidase (MAO), which is a mitochondrial marker enzyme, increased significantly in response to fenofibrate in the HF and LF diet groups (Fig. 6D). In contrast, MAO activity decreased significantly in muscle and increased in adipose tissue in the LF diet group treated with fenofibrate. The interaction effects between the lipid level and fenofibrate treatment were only observed in the MAO activities in liver and muscle (Supplemental Table S3).

### Nutritional background affects PPARα mRNA and protein expression

As shown in Fig. 7A, fenofibrate significantly increased PPARα mRNA level only in the HF diet group. The western blot analysis indicated an increase in PPARα protein level only in the HF group (Fig. 7B,C), indicating that fenofibrate efficiently activated PPARα in tilapia, but was largely affected by nutritional background.

### mRNA expression of lipid metabolism genes in liver, muscle, and adipose tissue change during the fenofibrate feeding trial

Figures 8, 9, 10 show the changes in mRNA expression of lipid metabolism genes in liver, muscle, and adipose tissue during the fenofibrate feeding trial. In general, most of the genes did not change significantly in either the LF or HF diet groups. Fenofibrate increased hepatic mRNA levels of the lipid transport gene FABP4 (Fig. 8F) in the HF diet group, but decreased ACCβ (Fig. 8D) mRNA expression levels in the LF diet group. Fenofibrate did not alter PPARα or PPARβ mRNA expression levels in muscle (Fig. 9A,B), but significantly increased CPT1a and CPT1b mRNA levels (Fig. 9C,D) in the HF diet group. Fenofibrate decreased PPARα mRNA expression in muscle (Fig. 9A) in the LF diet group. Fenofibrate had less of an effect on gene expression in adipose tissue compared with that in liver and muscle. In the adipose tissue, fenofibrate did not change PPARα mRNA expression (Fig. 10A) and slightly increased ATGL mRNA level (Fig. 10D) in the LF diet group. The interaction effects between the lipid level and fenofibrate treatment were only seen in the mRNA level of CD36 in liver and ATGL mRNA level in adipose tissue (Supplemental Table S3). These results suggest that fenofibrate stimulated activation of PPARα only in the liver of fish fed the HF diet and had a relatively weak stimulatory effect on lipid metabolism-related gene expression in the three tissues.

## Discussion

### Activation of PPARα and lipid metabolism in different species fed with HF diet

The effects of the HF diet on PPARα activation and the expression of lipid metabolism-related genes have been studied in many species. PPARα mRNA expression is stimulated by the HF diet in mice, particularly the liver, which triggers up-regulation of a number of PPARα target genes, such as CPT1 and FABPs, to enhance fat utilization and counteract excessive lipid loading^25^,^26^,^27^,^28^. One study reported that a HF diet increased hepatic fatty acid oxidation by 68% in mice^13^. A similar change was reported in macaque^29^. Feeding a HF diet results in excess circulating and stored unesterified FFAs^8^,^27^, which is the main cause of lipotoxicity^30^,^31^. However, FFAs are also endogenous PPARα ligands^32^, and a number of *in vitro* and *in vivo* studies have reported that PPARα is activated by FFAs, which consequently increases the expression of downstream genes involved in lipid breakdown^33^,^34^. Therefore, PPARα in HF diet-fed mammals could be activated by high concentrations of FFAs and stimulate lipid breakdown. This has been described as a self-protection mechanism in mammals to alleviate the toxic effects of lipid overload. In the first phase of the present study, feeding the HF diet did not increase mRNA expression of PPARα and other genes involving in lipid catabolism or PPARα-regulated downstream pathways. In addition, the HF diet did not result in severe fat accumulation in liver or excess plasma FFA concentrations but did cause higher fat deposition in mesenteric tissue compared with those in the LF diet group. Our previous study indicated that HF diet-fed Nile tilapia increases uptake of FFAs and enhances TG synthesis in adipose tissue, accompanied by increased intracellular lipolysis that releases FFAs to activate PPARγ and trigger adipocyte proliferation to maintain lipid homeostasis^35^. It should be noticed that the results of this experiment were obtained in the fed state^35^, whereas all samples of the present experiment were collected from 24 h-fasting state. However, in the HF dietary group in the present study, the higher SREBP1c, ATGL, and HSL mRNA expression levels in adipose tissue confirmed our previous results and demonstrated the regulatory function of adipose tissue in Nile tilapia to maintain lipid homeostasis and stable serum FFA concentration during HF diet feeding. As another proof, FABP4, which is an important binding protein for intracellular FFA transport, was down-regulated in liver and muscle. These results suggest that circulating FFA concentrations and those in most tissues observed during the present 10-week HF feeding trial may not be sufficient to activate PPARα or its target genes. Some studies have indicated that the activating sensitivity of PPARα varies among species, such as PPARα induction in the human liver is less sensitive than that in mice^36^. Our recent study paper also suggested the activating sensitivity of PPARα in tilapia is relatively weak, compared with that in rodents^37^. Taken together, PPARα and PPARα-triggered lipid breakdown may not be sufficiently activated by high lipid intake in fish, suggesting that the self-protective mechanism of fish in response to high energy intake has not been well established from an evolutionary perspective.

### Nutritional background changes the hypolipidemic effects of fenofibrate

Fibrates, including fenofibrate and clofibrate, are well-known pharmacological PPARα agonists widely used to treat dyslipidemia. The functions of fibrates in mammals include reducing plasma TG level^38^, improving nonalcoholic fatty liver disease^39^, and preserving insulin signal transduction in mice^13^. Many animal studies have demonstrated significant lipid-lowering functions of fibrates under the HF diet feeding condition, which mimics the Western human dietary pattern^40^,^41^. The strong effect of fibrates on reducing intrahepatic TG content in obese mammalian models has been widely reported^42^,^43^,^44^. In contrast, the significant effect of fibrates would be abolished when animals are fed a chow diet^45^. In fact, one study reported that fenofibrate activated PPARα and the lipid anabolic gene SREBP1c in mice fed a normal diet^46^, suggesting that the lipid-lowering effect of fenofibrate is attenuated by the enhanced lipid synthesis caused by low dietary lipid intake. Similarly, an increase in lipid synthesis has also been reported in Nile tilapia fed a LF diet^35^. In the present study, feeding the HF diet during the first phase did not activate PPARα in tilapia, suggesting low sensitivity to PPARα induction in tilapia. Therefore, we tested the effect of fenofibrate and the possible effects of nutritional background on activation of PPARα during the second phase. Although the two-way ANOVA did not show many interactions between dietary lipid level and fenofibrate treatment as two factors, the hypolipidemic effects of fenofibrate differed between HF and LF dietary groups. The results indicated that fenofibrate significantly increased PPARα mRNA and protein expression levels in the tilapia fed the HF diet, accompanied by reduced liver and plasma TG concentrations and increased CPT1a/CPT1b mRNA expression in muscle. This result also partly agrees with a previous study on yellow catfish that dietary fenofibrate supplementation increases PPARα expression and decreases hepatic and plasma TG levels in fish with excess hepatic lipid deposition induced by zinc^22^. In the present study, PPARα, CPT1, and SREBP1c expression did not increase in response to fenofibrate in the LF diet-fed group, whereas fenofibrate increases PPARα and SREBP1c expression in mice fed a normal diet^46^, suggesting again that the activating sensitivity of PPARα in tilapia is relatively weak. As a proof, we tested the mRNA expression of PPARα-sensitive downstream genes (PDK4 and Plin2) in primary tilapia hepatocytes exposed to 200 nM fenofibrate for 24 h, but none of them was affected (Supplemental Fig. 2C). In addition, we noticed that the dose of fenofibrate with 60 mg/kg body weight could induce significantly lipid-lowering effect in yellow catfish^19^, and 100 mg/kg body weight could also induce some biochemical alteration in lipid metabolism in grass carp^20^ and rainbow trout^47^. In the present study, the fenofibrate dose of 200 mg/kg body weight is higher than the dose used in other fish. This indicates again that the activation of PPARα in Nile tilapia is relatively weak. Interestingly, hepatic FA β-oxidation efficiency and MAO activities in the LF and HF diet groups both increased after the fenofibrate treatment (Fig. 6), suggesting that fenofibrate stimulates hepatic fatty acid β-oxidizing activity, regardless of nutritional background, which was partly caused by increased cellular mitochondrial density. This finding partly agrees with results obtained from monkeys that dietary supplementation with fenofibrate significantly increases mitochondrial proliferation but only moderately changes mRNA expression of most oxidation pathway proteins^47^. Monkeys and humans are regarded as less sensitive to PPARα activation compared with that in rodents^7^,^36^,^48^, which is similar to the tilapia result in the present study. These findings suggest that fenofibrate can induce proliferation of mitochondria and increase mitochondrial FA β-oxidation efficiency in animals with relatively low sensitivity to PPARα activation. As the proof, in the present study, higher FA β-oxidation was measured either in the LF or HF dietary groups after fenofibrate treatment when [1-^14^C] palmitic acid was provided in sufficient quantity in the *in vitro* measurement. However, the hypolipidemic effects of fenofibrate differed in the *in vivo* situation. This could be explained that in the fish fed with HF-fenofibrate diet, excess fat intake would cause high concentration of circulating FFA and then the FFA as substrates would be degraded by mitochondria in which the FA β-oxidation activity was elevated through PPARα activation. Therefore, fenofibrate played significantly lipid-lowing effects in HF diet-feeding fish. However, in the fish fed with LF-fenofibrate diet, the insufficient lipid intake caused fish preferentially to increase the lipogenesis to maintain lipid homeostasis, thus the circulating FFA would preferentially enter esterification pathway but not mitochondrial FA β-oxidation. In fact, our previous study also indicated that even glycolysis is up-regulated to produce more acetyl-CoA to satisfy the lipogenesis requirement in LF diet-fed tilapia^35^. Therefore, the lipid-lowing effects of fenofibrate could not be observed in the LF dietary group, even if the activity of mitochondrial FA β-oxidation was increased. Actually, in our pre-experiment, we measured the [1-^14^C] labeled CO_2_ in the fenofibrate-treated tilapias which were intraperitoneally injected with high or low dose of [1-^14^C] palmitate, we found higher content of palmitate injection caused higher amount of CO_2_ production (Supplemental Fig. 3), showing that the lipid catabolism rate is tightly correlated to the substrate concentration. Taken together, nutritional background, including fat deposits and dietary lipid content, may change the effects of PPARα ligands, such as fenofibrate, by affecting substrate availability for lipid catabolism. However, it is of note that the mRNA changes of the most genes assayed in the present study do not directly reflect the enzyme activities or protein functions, therefore, the molecular regulatory mechanisms of fenofibrate at the transcriptional level still need further functional validation.

## Conclusions

PPARα is an important transcriptional regulator of genes involved in lipid metabolism and is activated by HF diet feeding and fibrates in mammals. However, our present study verified that HF diet feeding did not activate PPARα in Nile tilapia. We also illustrated that fenofibrate activated hepatic PPARα expression and played a hypolipidemic effect only under the HF feeding condition, but not in the Nile tilapia fed the LF diet. This result was related to different substrate availability for lipid catabolism under the two nutritional backgrounds. This is the first study to show that physiological fat deposition and dietary lipid content change activation of PPARα and the hypolipidemic effects of fenofibrate in fish and could be a reference for other species.

## Materials and Methods

### Feeding trial and sampling

**Figure 1:**
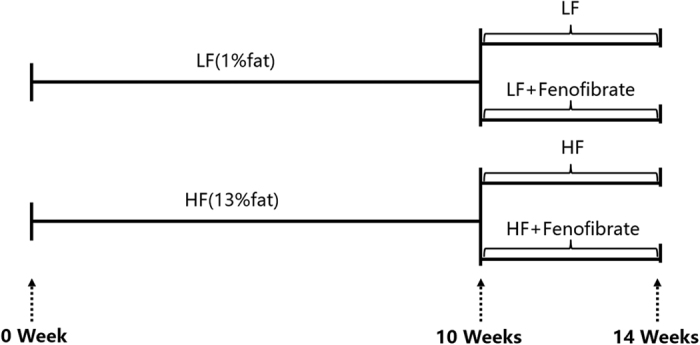
Experimental protocol of the two-phase feeding trial. HF, high-fat; LF, low-fat.

**Figure 2:**
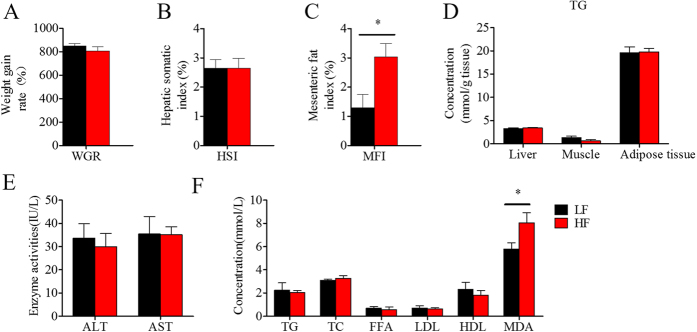
The growth, lipid deposition and biochemical parameters of the Nile tilapia fed with high or low fat diet. (**A**) Weight gain rate (WGR); (**B**) Hepatosomatic index (HSI); (**C**) Mesenteric fat index (MFI); (**D**) Triglyceride concentrations (TG); (**E**) Plasma activities of ALT and AST; (**F**) Plasma concentrations of TG, TC, FFA, LDL, HDL and MDA. Hepatosomatic index = liver weight × 100/fish weight; Mesenteric fat index = mesenteric fat tissue weight × 100/fish weight. Values are means ± SEM (n = 6). The difference between two diets was compared using t-test (*P < 0.05).

**Figure 3:**
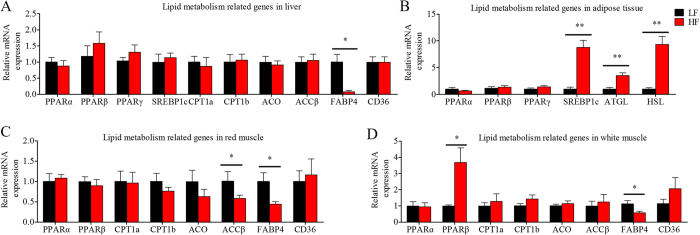
The mRNA expression of the genes related to lipid metabolism in the Nile tilapia fed with high or low fat diet. (**A**) The genes related lipid metabolism in liver; (**B**) The genes related lipid metabolism in adipose tissue; (**C**) The genes related lipid metabolism in red muscle; (**D**) The genes related lipid metabolism in white muscle. Values are means ± SEM (n = 6). The difference between two diets was compared using t-test (*P < 0.05 and **P < 0.01).

**Figure 4:**
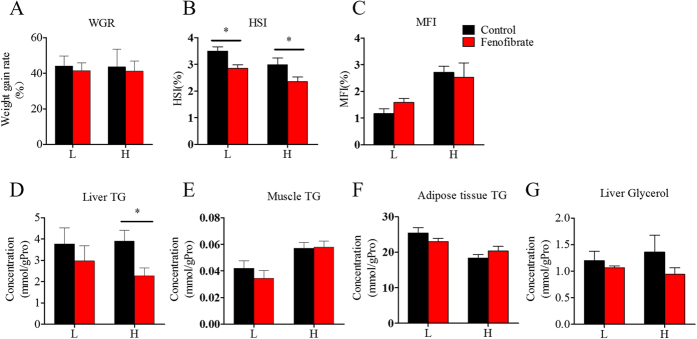
The effects of fenofibrate on the growth and lipid deposition in the Nile Tilapia fed with high or low fat diet. (**A**) Weight gain rate (WGR); (**B**) HSI; (**C**) Liver TG; (**D**) Liver glycerol; (**E**) Muscle TG; (**G**) Adipose tissue TG. Values are means ± SEM (n = 6). The difference between Fenofibrate and Control was compared using t-test (*P < 0.05).

**Figure 5:**
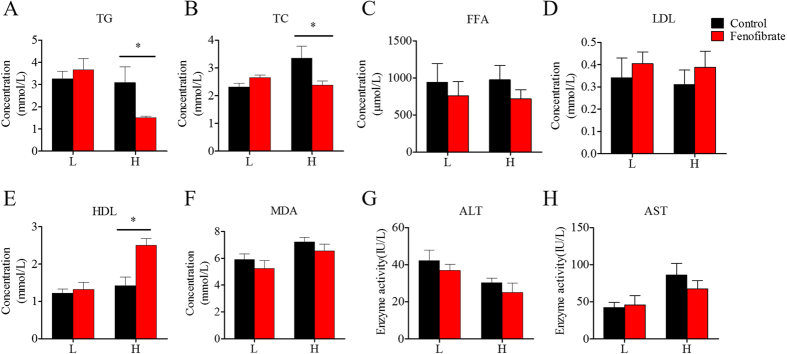
The effects of fenofibrate on the plasma biochemical parameters in the Nile Tilapia fed with high or low fat diet. (**A**) Plasma TG; (**B**) Plasma TC; (**C**) Plasma FFA; (**D**) Plasma LDL; (**E**) Plasma HDL; (**F**) Plasma MDA; (**G**) Plasma ALT; (**H**) Plasma AST. Values are means ± SEM (n = 6). The difference between Fenofibrate and Control was compared using t-test (*P < 0.05).

**Figure 6:**
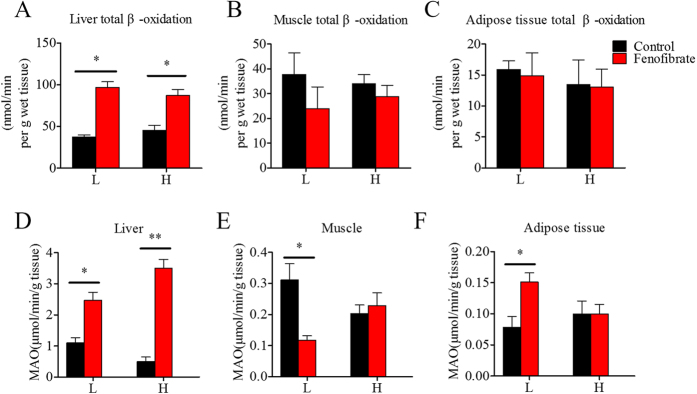
The effects of fenofibrate on the activities of fatty acid β-oxidation and monoamine oxidase (MAO) in tissues in the Nile tilapia fed with high or low fat diets. (**A**) Total β-oxidation of [1-^14^C] palmitic acid in liver; (**B**) Total β-oxidation of [1-^14^C] palmitic acid in muscle; (**C**) Total β-oxidation [1-^14^C] palmitic acid in adipose tissue; (**D**) MAO activity in liver; (**E**) MAO activity in muscle; (**F**) MAO activity in adipose tissue. Values are means ± SEM (n = 4). The difference between Fenofibrate and Control was compared using t-test (*P < 0.05 and **P < 0.01).

**Figure 7:**
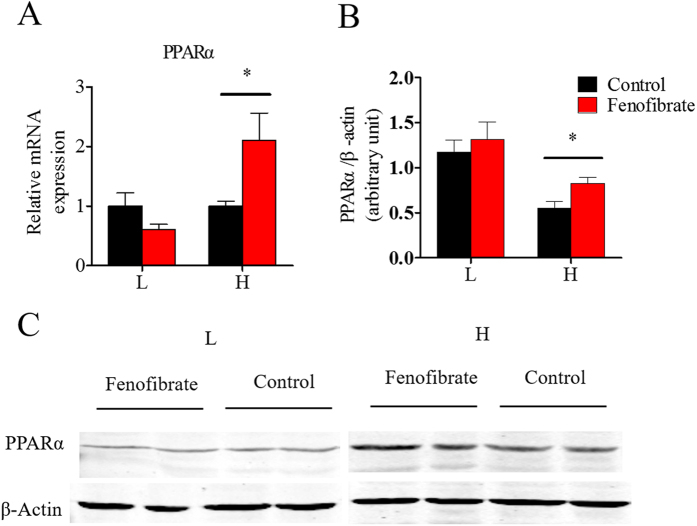
The effects of fenofibrate on the mRNA and protein expression of PPARα in the Nile tilapia fed with high or low fat diet. (**A**) The relative mRNA abundance of PPARα; (**B**) The relative quantitated result of WB of PPARα; (**C**) The result of western blotting, PPARα antibody was used(top) and β-actin antibody was used as a loading control (bottom). For A and B, values are means ± SEM (n = 6). The difference between Fenofibrate and Control was compared using t-test (*P < 0.05).

**Figure 8:**
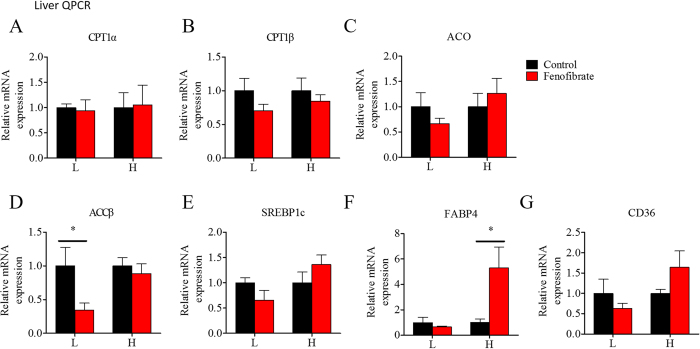
The effects of fenofibrate on the mRNA expression of the genes related to lipid metabolism in liver of the Nile tilapia fed with high or low fat diet. (**A–D**) The relative mRNA abundance of CPT1a, CPT1b, ACO and ACCβ showing the activity of FA β-oxidation; (**E**) The relative mRNA abundance of SREBP1c which plays important role in lipogenesis; (**F**) The relative mRNA abundance of FABP4 showing the activity of intracellular FA transport; (**G**) The relative mRNA abundance of CD36 showing the ability of FA uptake. Values are means ± SEM (n = 6). The difference between Fenofibrate and Control was compared using t-test (*P < 0.05).

**Figure 9:**
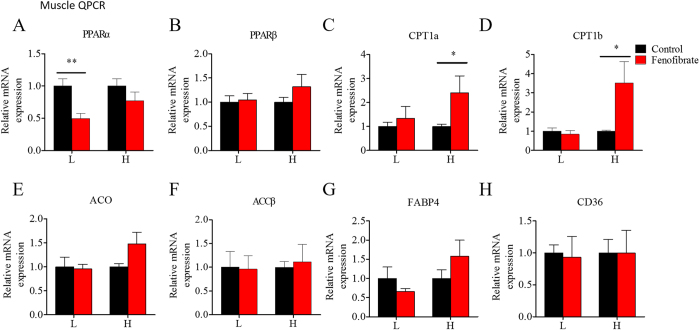
The effects of fenofibrate on the mRNA expression of the genes related to lipid metabolism in muscle of the Nile tilapia fed with high or low fat diet. (**A,B**) The relative mRNA abundance of PPARα and PPARβ; (**C–F**) The relative mRNA abundance of CPT1a, CPT1b, ACO and ACCβ showing the activity of FA β-oxidation; (**G**) The relative mRNA abundance of FABP4 showing the activity of intracellular FA transport; (**H**) The relative mRNA abundance of CD36 showing the ability of FA uptake. Values are means ± SEM (n = 6). The difference between Fenofibrate and Control was compared using t-test (*P < 0.05 and **P < 0.01).

**Figure 10:**
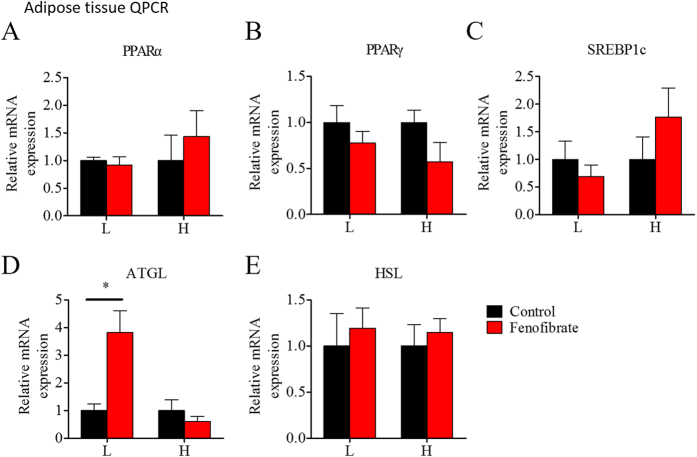
The effects of fenofibrate on the mRNA expression of the genes related to lipid metabolism in adipose tissue of the Nile tilapia fed with high or low fat diet. (**A–C**) The relative mRNA abundance of PPARα, PPARγ and SREBP1c; (**D,E**) The relative mRNA abundance of ATGL and HSL showing the activities of lipolysis. Values are means ± SEM (n = 6). The difference between Fenofibrate and Control was compared using t-test (*P < 0.05).

Nile tilapias were obtained from the Fishery Genetic Resources Experiment Station of Shanghai Ocean University (Shanghai, China). All of them were acclimated with commercial diets (Dajiang, China) for one month. The schedule of the formal two-phase feeding trial is shown in Fig. 1. The first feeding phase was started after the acclimation. One hundred and sixty-eight fish weighed average 2.24 ± 0.04 g were randomly distributed into six glass tanks (200 liters; twenty-eight fish per tank, 3 tanks per dietary group). The fish were assigned with two iso-proteic diets (43.2% protein) containing 1 or 13% lipid levels (LF and HF), respectively. The formulation of the diets is presented in the supplemental Table S1, and the diets were made as described previously^35^. The first phase feeding trial lasted for ten weeks at a feeding rate of 4% BW/d to induce different background of body fat accumulation. At the end of the first phase feeding trial, the growth between two groups were comparable, thus 24 Nile tilapia with similar body weight (22.29 ± 0.70 g) were selected from two dietary groups, respectively, for the second phase feeding trial. The fish selected from the same dietary group were then divided to two tanks (12 fish/tank) and fed with the same diet as in the first phase, but the diet of one group was supplemented with fenofibrate, the PPARα ligand. The fenofibrate purchased from the Sigma Chemical Co. (St. Louis, MO) was mixed into the HF or LF diet to the final dose of 200 mg fenofibrate/kg BW per d when the feeding rate was set as 4% BW, the formulation of the diets is presented in the supplemental Table S1. In short, in the second feeding phase, the fish in the four tanks were fed with four diets as LF, LF + fenofibrae, HF and HF + fenofibrate, respectively, for four weeks. To precisely track the growth of every fish, each fish was embedded with a tracking tag with a unique Radio-frequency identification (RFID) code, which could be recognized by a RFID machine (Boise, ID, USA) as described previously^49^. During the feeding trials, fish were fed at 9:00 and 18:00 with an equal portion of diet, and the weight of each fish was recorded and tracked at each week. Water temperature was maintained at 28 ± 1 °C with a 12 h light-dark cycle for 4 weeks. At the end of trial, all fish were 24 h fasted, six fishes of each group were euthanized (MS-222 at 20 mg/l) and sampled to collect tissues to measure the molecular, protein or biochemical indexes. All experiments were conducted under the Guidance of the Care and Use of Laboratory Animals in China. This research was approved by the Committee on the Ethics of Animal Experiments of East China Normal University.

### Catabolic rate assay of the intraperitoneally injected [1-^14^C] palmitate in living fish

To mimic the catabolism in the different substrate concentrations, a pre-experiment was performed as following procedure: eight fenofibrate-treated fishes with similar body weight (50 g ± 2 g) were divided as two groups: LPA group (received an intraperitoneal injection of 20 μl DMSO containing 20 nM [1-^14^C] palmitate with 0.2μCi per 50 g BW) and HPA group (received an intraperitoneal injection of 20 μl DMSO containing 50 nM [1-^14^C] palmitate with 0.5 μCi per 50 g BW). The injected fish was immediately moved to a closed and oxygen-saturated water-contained glass jar, which was connected with another glass bottle containing saturated KOH solution. The details of the experimental process were described previously^50^,^51^. The KOH solution containing the [1-^14^C] carbon dioxide sourced from the breakdown of the [1-^14^C] palmitate was collected at 2, 25 and 40 min. The radioactivity of the KOH was measured after mixing with the scintillation cocktail medium Ultima Gold XR (Perkin, USA) in a liquid scintillation spectrometer MicroBeta Trillux 1450 (Perkin, USA).

### Biochemical assays

Hepatic triglyceride (TG) and glycerol and plasma TG, glucose and lactate were assessed by the commercial kits (Jiancheng Biotech Co., China). The plasma free fatty acid (FFA) was measured by ELISA kits (R&D Systems, USA). The abundance of mitochondria in tissue was assessed by measuring the activity of monoamine oxidase (MAO), the marker enzyme of mitochondria, in tissue homogenate as previously indicated^52^.

### [1-^14^C] palmitate oxidation in liver, muscle and adipose tissue homogenates

At the end of the second phase, pieces of liver, muscle and adipose tissue (about 0.2 g) collected from each group were cut finely in ice-cold 0.25 M-sucrose medium containing 2 mM-EGTA and 10 mM-2-amino-2-hydroxymethyl-propane-1,3-diol-HCl, pH 7.4, rinsed five times in the same medium, blotted with absorbent paper and weighed. The tissues were respectively diluted (1:40, 1:20 and 1:10, w/v) in the chilled sucrose medium and homogenized by using a drill-driven Teflon glass homogenizer (Ningbo Scientz Biotechnology co., China) with 4-6 strokes. The 1 ml samples of homogenate were used for the immediate measurement of [1-^14^C] palmitate oxidation^20^,^47^. Palmitate oxidation rate was measured at 28 °C using a media allowing both mitochondrial and peroxisomal FA oxidation to occur as already described^53^. After 30 min, the reaction was stopped by addition of 10% (w/v) perchloric acid, which precipitated proteins. The media were filtered using Millipore filters (0.45 μm pore size) under very low pressure and the filtrate containing the acid-soluble products (ASP, the short metabolites from FA oxidation) was mixed with Ultima Gold XR (Packard) for radioactivity measurements.

### Quantitative real time PCR and western blot analyses

RNA isolation, cDNA synthesis and quantitative PCR were performed as described previously^54^. Primer details are provided in the supplemental Table S2. Quantitative PCR efficiency was between 98% and 102% and the correction coefficient was over 0.97 for each gene. Each PCR run performed in triplicate and negative controls (no cDNA) were conducted. The relative cDNA abundance was estimated as the 2^−ΔΔCt^ method (control group as control), thereof, ΔCt = Ct_target_ − (Ct_EF1α_ + Ct_β-actin_)/2.

The antibody against rabbit PPARα (Proteintech, USA), antibody against rabbit β-Actin (Huabio, China) and goat anti-rabbit IgG (Li-cor, USA) were used. Preliminary experiment was conducted to choose an appropriate antibody against peroxisome proliferator-activated receptor alpha (PPARα). Finally, a rabbit polyclonal antibody against mouse PPARα from Proteintech (Catalog no. 15540-1-AP; Proteintech Group, Inc., Chicago, IL) was chosen. The procedure of manipulation was performed as described in He *et al*.^35^. The detection was achieved using the Odyssey CLx Imager (Li-cor, USA). This western blotting experiment was repeated for three times.

### Statistical analysis

Results are expressed as mean ± SEM. Independent-samples t-test was performed to evaluate the significant difference (P < 0.05) of variables between high and low diet lipid level of first phase feeding trial or control and fenofibrate treatment of second phase feeding trial. Two-way ANOVA analysis was used to explore the possible interactions existing between lipid level and fenofibrate treatment of second phase feeding trial. All analyses were conducted using the IBM SPSS Statistics 21 (IBM, USA).

## Additional Information

**How to cite this article**: Ning, L.-J. *et al*. Nutritional background changes the hypolipidemic effects of fenofibrate in Nile tilapia (*Oreochromis niloticus*). *Sci. Rep.* **7**, 41706; doi: 10.1038/srep41706 (2017).

**Publisher's note:** Springer Nature remains neutral with regard to jurisdictional claims in published maps and institutional affiliations.

## Supplementary Material

Supplemental Figures and Tables
