# Supplementary material for: Nutritional background changes the hypolipidemic effects of fenofibrate in Nile tilapia (Oreochromis niloticus)
Source: Sci Rep. 2017 Jan 31;7:41706. doi: 10.1038/srep41706 (PMC5282496; doi:10.1038/srep41706)
Supplement: Supplemental Figures and Tables [file srep41706-s1.pdf]

Nutritional background changes the hypolipidemic effects of fenofibrate in Nile tilapia (*Oreochromis niloticus*)

Li-Jun Ning, An-Yuan He, Dong-Liang Lu, Jia-Min Li, Fang Qiao, Dong-Liang Li, Mei-Ling Zhang, Li-Qiao Chen, Zhen-Yu Du*

Laboratory of Aquaculture Nutrition and Environmental Health (LANEH), School of Life Sciences, East China Normal University, Shanghai, China

Corresponding author:

Prof. Zhen-Yu Du

Laboratory of Aquaculture Nutrition and Environmental Health (LANEH), School of Life Sciences, East China Normal University, Shanghai 200241, PR China

E-mail address: zydu@bio.ecnu.edu.cn;

Telephone: + 86-21-54345354

Supplemental information

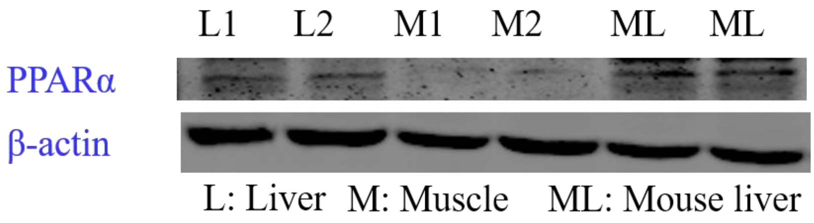

Supplement Figure S1

Evaluation of the target protein specificity of PPARα antibodies in the liver and muscle of tilapia and the liver of mouse.

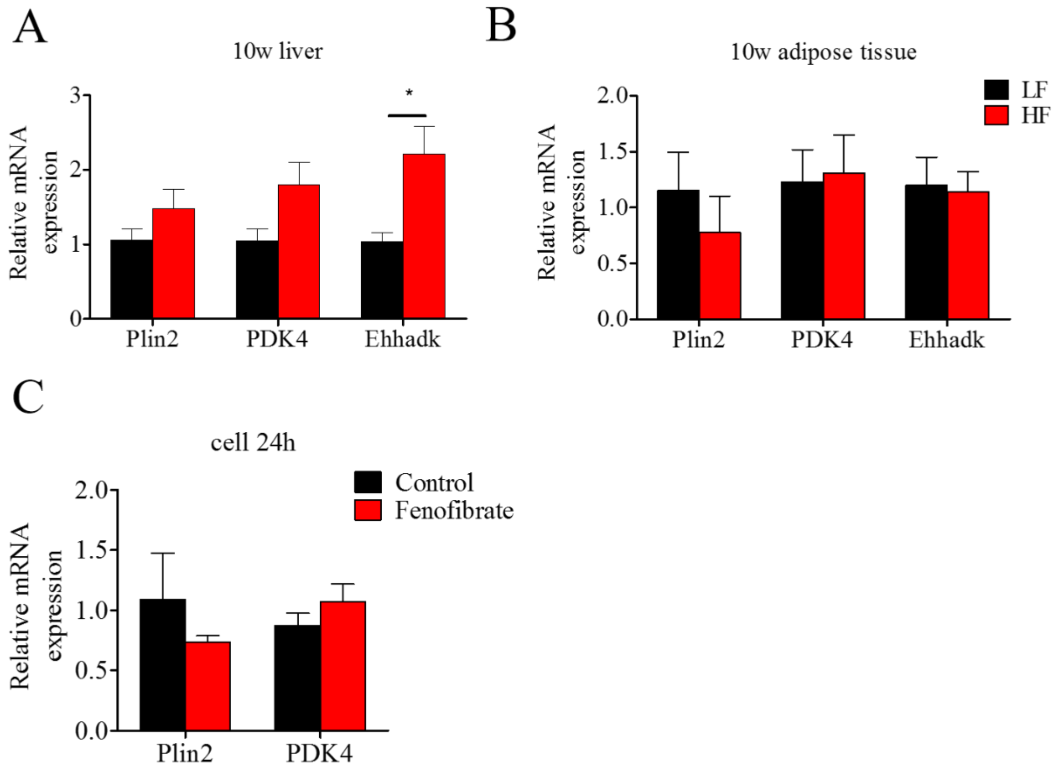

Supplement Figure S2

The mRNA expression of PPARα-sensitive downstream genes in liver, adipose tissue and primary hepatocytes of Nile tilapia. A) The mRNA expression of Plin2, Pdk4 and Ehhadh in liver of the Nile tilapia fed with high or low fat diet; B) The mRNA expression of Plin2, Pdk4 and Ehhadh in adipose tissue of the Nile tilapia fed with high or low fat diet; C) The mRNA expression of Plin2 and Pdk4 in primary hepatocytes of the Nile tilapia treat with fenofibrate. Values are means ± SEM (n = 6). The difference between HF and LF or Fenofibrate and Control was compared using t-test (*P < 0.05).

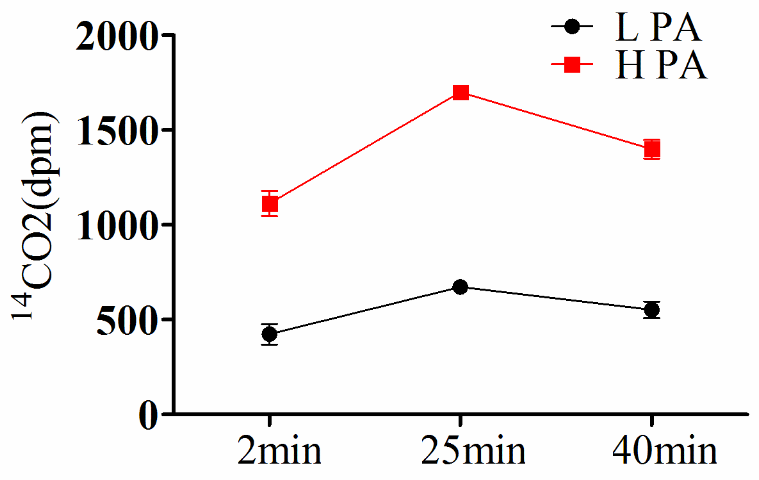

Supplement Figure S3

The assay of the catabolism rate of intraperitoneal injected [1-^{14}C] palmitate in fenofibrate-treated Nile tilapia via collecting [1-^{14}C] CO2 at different time points. LPA, low dose of [1-^{14}C] palmitate (20 nM); HPA, high dose of [1-^{14}C] palmitate (50 nM).

Supplemental Table S1.

Formulation and proximate composition of the experimental diets

Component (g/kg)	LF	HF
Casein	360	360
Gelatin	80	80
Soybean oil	10	130
Corn starch	324.75	324.75
Vitamin premix[1]	10	10
Mineral premix[2]	40	40
CMC	30	30
Cellulose	140	20
Choline chloride	5	5
BHT	0.25	0.25
Total	1000	1000
Proximate Composition		
Dry matter (%)	92.31	92.25
Crude protein (%)	43. 21	43.25
Crude lipid (%)	1.17	13.11
Ash (%)	5.23	5.16

[1]Vitamin premix, (mg or IU/kg): 500,000 I.U. (international units) Vitamin A, 50,000 I.U. Vitamin D3, 2500 mg Vitamin E, 1000 mg Vitamin K3, 5000 mg Vitamin B1, 5000 mg Vitamin B2, 5000 mg Vitamin B6, 5000 μg Vitamin B12, 25,000 mg Inositol, 10,000 mg Pantothenic acid, 100,000 mg Cholin, 25,000 mg Niacin, 1000 mg Folic acid, 250 mg Biotin, 10,000 mg Vitamin C.

[2]Mineral premix, (g/kg): 314.0 g $CaCO_3$; 469.3 KH_2PO_4; 147.4 g $MgSO_4 \cdot 7H_2O$; 49.8 g NaCl; 10.9 g Fe(II) gluconate; 3.12 g $MnSO_4 \cdot H_2O$; 4.67 g $ZnSO_4 \cdot 7H_2O$; 0.62 g $CuSO_4 \cdot 5H_2O$; 0.16 g KJ; 0.08 g $CoCl_2 \cdot 6H_2O$; 0.06 g NH_4 molybdate; 0.02 g $NaSeO_3$.

Supplemental Table S2.

Primers used in quantitative PCR

Usage	Item	Primer name	Sequence (5'–3')	Size (bp)	Notes
RT-PCR	PPARα	q1f	AACCGAAACAAGTGCCAGTA	121	KF871430
cDNA		q1r	TTGCTTTCCTCCTTGAGCTT		
	PPARβ	q1f	AACCGAAACAAGTGCCAGTA	121	KF871430
		q1r	TTGCTTTCCTCCTTGAGCTT		
	PPARγ	Gq1F	TGGACTACACAAACATGCACAGC	101	KF918712
		Gq1R	CACGGGACTATCTGAGTACTGTGGA		
	SREBP1c	S1F	ATTCAGACTCAGCTCCAAGGG		
		S1R	TCAGGCTTCAAAGTGGTCAG		
	CPT1a	Ca2F	GTCCCATGGAAAAATGAGTACATCA	100	XM_005470876
		Ca2R	GGTAAGGAGGCCTGGAAACTGTA		
	CPT1b	C4F	AAGGGACGTTACTTCAAGGTG	100	GQ395696
		C4R	TCCGACTTGTCTGCCAAGAT		
	ACO	Acq1F	AGTCCCACTGTGAGCTCCATCAA	108	KF918710
		Acq1R	CAGACCATGGCAGTTTCCAAGA		
	ACCβ	Ab2F	ACATGCAGTCCATGCTGCGT		XM_003451659
		Ab2R	AAATGCCTCTCAAGCCACTCAA		
	FABP4	Fa1F	AAGCTGGGAGAGGAGTTTGATGAA	112	
		Fa1R	TCTCTTTGCCGTCCCACTTCT		
	CD36	CD2F	TCACCTGAAAGGACACATCACCA	104	XM_003451528
		CD2F	CGCTGCATCAAGATGTTCACCT		
	HSL	h1f	TTCCCGTCTGCTAACACTC	108	FJ601660
		h1r	TTCTCCACCTGCTTCTCG		
	ATGL	ATmF	CCCGAGTTACAGACGGAGAA	104	
		ATmR	AGGAGGAATGATGCCACAG		
	PDK4	P1F	GGTTATGGCACAGCAGCGGTCA	119	XM_003457260.3
		P1R	TCATCGGCCTCTGTGGTGGACT		
	Plin2	Pn1F	TGCTTCACCCATCATCGACA	95	KF751704
		Pn1R	TCGGCAGGGTTTTCTCAATC		
	Ehhadh	Eh1F	AGTCAGTGTGGCGGTTGGCAAT	107	XM_003447428.3
		Eh1R	TCAGGTGTGGCTCCCTCTTCCA		
	EF1α	E1F	CTACGTGACCATCATTGATGCC	106	KJ123689
		E1R	AACACCAGCAGCAACGATCA		
	β-actin	Ba1F	CAGGATGCAGAAGGAGATCACA	92	KJ126772
		Ba1R	CGATCCAGACGGAGTATTTACG		

Supplemental Table S3.
The results of two-way ANOVA analysis of Figure 4-10.

		One-way ANOVA	Two-way ANOVA			
**Figure 4**	Growth and lipid deposition	Significance (P value)		Lipid level (L)	Fenofibrate treatment (F)	L ×F
Fig. 4A	WGR	0.982	*F*	0.527	1.264	0.555
			P	0.481	0.281	0.470
Fig. 4B	HSI	*0.041*	*F*	5.044	12.144	0.125
			P	0.043	0.004	0.729
Fig. 4C	MFI	*0.006*	*F*	10.022	0.01	4.405
			P	0.007	0.92	0.056
Fig. 4D	Liver TG	*0.167*	*F*	0.113	0.736	0.649
			P	0.742	0.406	0.435
Fig. 4E	Muscle TG	*0.029*	*F*	10.357	0.337	0.510
			P	0.007	0.572	0.488
Fig. 4F	Adipose tissue TG	*0.005*	*F*	1.339	0.172	2.033
			P	0.268	0.685	0.177
Fig. 4G	Liver Glycerol	*0.461*	*F*	0.349	0.001	0.924
			P	0.565	0.980	0.354
**Figure 5**	Plasma biochemical parameters			Lipid level (L)	Fenofibrate treatment (F)	L ×F
Fig. 5A	TG	*0.072*	*F*	6.134	2.229	4.744
			P	0.24	0.154	0.044
Fig. 5B	TC	*0.016*	*F*	0.005	0.074	1.163
			P	0.947	0.789	0.296
Fig. 5C	FFA	*0.737*	*F*	0.581	0.091	0.473
			P	0.456	0.767	0.501
Fig. 5D	LDL	*0.798*	*F*	0.003	0.009	0.025
			P	0.958	0.924	0.875
Fig. 5E	HDL	*0.014*	*F*	0.222	0.836	0.352
			P	0.644	0.373	0.561
Fig. 5F	MDA	*0.084*	*F*	2.425	0.765	1.230
			P	0.138	0.394	0.283
Fig. 5G	ALT	*0.055*	*F*	3.521	0.118	0.845
			P	0.078	0.735	0.371
Fig. 5H	AST	*0.061*	*F*	3.514	1.021	1.574
			P	0.078	0.327	0.227
**Figure 6**	FA β-oxidation			Lipid level (L)	Fenofibrate treatment (F)	L ×F

Fig. 6A	Liver	*0.000*	*F*	0.023	68.340	2.040
			P	0.883	0.000	0.191
Fig. 6B	Muscle	*0.537*	*F*	0.008	1.937	0.397
			P	0.932	0.201	0.546
Fig. 6C	Adipose tissue	*0.911*	*F*	0.464	0.045	0.011
			P	0.515	0.837	0.918
	MAO Activity					
Fig. 6D	Liver	*0.000*	*F*	0.930	94.421	13.210
			P	0.363	0.000	0.007
Fig. 6E	Muscle	*0.032*	*F*	0.001	5.497	9.294
			P	0.971	0.047	0.016
Fig. 6F	Adipose tissue	*0.081*	*F*	0.760	4.504	4.504
			P	0.409	0.067	0.067
**Figure 7**	mRNA and protein expression of PPARα			Lipid level (L)	Fenofibrate treatment (F)	L ×F
Fig. 7A	PPARα mRNA	*0.028*	*F*	0.360	0.196	0.160
			P	0.609	0.701	0.728
Fig. 7B	PPARα protein	*0.011*	*F*	46.840	8.354	1.554
			P	0.021	0.102	0.339
**Figure 8**	Liver QPCR			Lipid level (L)	Fenofibrate treatment (F)	L ×F
Fig. 8A	CPT1α	*0.995*	*F*	0.063	0.505	0.000
			P	0.814	0.516	0.986
Fig. 8B	CPT1β	*0.436*	*F*	0.001	0.137	1.299
			P	0.981	0.731	0.318
Fig. 8C	ACO	*0.516*	*F*	0.029	0.289	0.745
			P	0.874	0.619	0.437
Fig. 8D	SREBP1c	*0.091*	*F*	3.431	0.209	2.689
			P	0.138	0.671	0.176
Fig. 8E	ACCβ	*0.037*	*F*	0.256	0.103	0.087
			P	0.639	0.763	0.783
Fig. 8F	FABP4	*0.023*	*F*	0.704	0.423	0.899
			P	0.449	0.551	0.397
Fig. 8G	CD36	*0.110*	*F*	0.026	1.863	11.970
			P	0.879	0.244	0.026
**Figure 9**	Muscle QPCR			Lipid level (L)	Fenofibrate treatment (F)	L ×F
Fig. 9A	PPARα	*0.008*	*F*	1.339	5.090	0.029
			P	0.299	0.074	0.872
Fig. 9B	PPARβ	*0.488*	*F*	0.923	0.437	0.958

			P	0.381	0.538	0.373
Fig. 9C	CPT1α	*0.104*	*F*	0.181	1.795	0.013
			P	0.688	0.238	0.914
Fig. 9D	CPT1β	*0.002*	*F*	6.309	3.971	2.932
			P	0.054	0.103	0.147
Fig. 9E	ACO	*0.132*	*F*	0.120	0.015	0.177
			P	0.744	0.907	0.691
Fig. 9F	ACCβ	*0.987*	*F*	2.232	1.426	0.434
			P	0.195	0.286	0.539
Fig. 9G	FABP4	*0.200*	*F*	2.187	1.392	1.809
			P	0.199	0.291	0.236
Fig. 9H	CD36	*0.996*	*F*	0.878	0.000	0.547
			P	0.392	0.992	0.493
**Figure10**	Adipose tissue QPCR			Lipid level (L)	Fenofibrate treatment (F)	L ×F
Fig. 10A	PPARα	*0.728*	*F*	0.001	1.486	1.476
			P	0.978	0.277	0.278
Fig. 10B	PPARγ	*0.304*	*F*	0.016	0.044	4.878
			P	0.904	0.842	0.078
Fig. 10C	SREBP1c	*0.306*	*F*	1.156	0.095	1.337
			P	0.331	0.771	0.300
Fig. 10D	ATGL	*0.000*	*F*	0.437	6.870	13.273
			P	0.538	0.047	0.015
Fig. 10E	HSL	*0.914*	*F*	1.391	0.292	1.081
			P	0.291	0.612	0.346
